# LncRNA–miRNA–mRNA Regulatory Network Reveals Potential Immune Responses in Larval Tomato Hind (*Cephalopholis sonnerati*) Infected with RGNNV

**DOI:** 10.3390/biology15161379

**Published:** 2026-08-12

**Authors:** Xiaoli Guo, Chengbin Gao, Zhangfan Chen, Sheng Lu, Lei Wang, Wensheng Li, Xinlei He, Chuanjun Yang, Jianwei Li, Songlin Chen

**Affiliations:** 1 College of Life Sciences, Qingdao University, Qingdao 266071, China; 2 State Key Laboratory of Mariculture Biobreeding and Sustainable Goods, Yellow Sea Fisheries Research Institute, Chinese Academy of Fishery Sciences, Qingdao 266071, China; 3 Laizhou Mingbo Aquatic Product Co., Ltd., Yantai 261418, China; 4 Qingdao Institute of Blue Seed Industry, Qingdao 266071, China

**Keywords:** red-spotted grouper nervous necrosis virus (RGNNV), lncRNA–miRNA–mRNA, brain, immune response, *Cephalopholis sonnerati*

## Abstract

The red-spotted grouper nervous necrosis virus (RGNNV) exhibits high pathogenicity in larval *C. sonnerati*, yet the immune molecular mechanism remains unclear. Non-coding RNAs (ncRNAs) are vital in the host’s immune responses during viral infection. However, there has been no study on ncRNA research for this species to date. We systematically identified 105 DE microRNAs (miRNAs), 157 DE long non-coding RNAs (lncRNAs) and 31 DE circular RNAs between the infection group and control group. Functional enrichment analysis revealed that these differentially expressed genes were significantly enriched in pathways associated with innate immune defense, inflammatory, and cell death, such as JAK-STAT signaling pathway, NF-κB signaling pathway, apoptosis, and necroptosis. Furthermore, the lncRNA–miRNA–mRNA interaction network involving miR-93 was constructed, which may represent a promising candidate therapy target for future investigations. This study presents the first comprehensive ncRNA transcriptome dataset of *C. sonnerati* infected with RGNNV, identifies key antiviral defense and cell death-related genes and hub pathways, and thereby identifies miR-93-involved lncRNA–miRNA–mRNA network and key targeted genes (*STAT1, TRIM25, UNC93B, IL12RB1, IRF8, CDKN1A, FCGR1A*) as hub molecular regulators in immune response of this species.

## Simple Summary

*Cephalopholis sonnerati* is an economically new aquaculture species in China. The red-spotted grouper nervous necrosis virus (RGNNV) exhibits high pathogenicity in larval *C. sonnerati*, but its immune response mechanisms remain unexplored, impeding the development of aquaculture. In the present study, we profiled brain transcriptome from healthy and naturally RGNNV-infected larvae. Many differentially expressed microRNAs (miRNAs), long non-coding RNAs (lncRNAs) and fewer circular RNAs were identified. Enrichment analysis demonstrated that these targeted genes of differentially expressed ncRNAs were markedly enriched in innate immune defense, inflammatory, and cell death related pathways, such as JAK-STAT signaling pathway, NF-κB signaling pathway, apoptosis, and necroptosis. Furthermore, a lncRNA–miRNA–mRNA regulatory network focused on miR-93 was established, which may provide a valuable candidate target for future antiviral strategies in *C. sonnerati.* This study offers the first ncRNA transcriptome landscape of *C. sonnerati* during RGNNV infection, establishing a theoretical basis for elucidating host-RGNNV interaction mechanism in groupers.

## 1. Introduction

Tomato hind (*Cephalopholis sonnerati*), belonging to the genus *Cephalopholis* in the Serranidae family, is mainly distributed in tropical and subtropical waters of the Indo-Pacific [1,2,3]. As a newly emerging aquaculture species, it holds considerable economic promise and substantial farming potential, owing to its distinctive bright red coloration, superior flesh quality, and exceptional nutritional composition, which includes a full spectrum of amino acids and abundant gelatinous components. During our large-scale breeding efforts conducted in Laizhou, Shandong Province, naturally large-scale mortality events were observed in *C. sonnerati* larvae. According to official OIE reports, VNN was confirmed as the most significant fish health challenge in Mediterranean region and the most important threat to the wild and cultivated population in South-East Asia [4].

The nervous necrosis virus (NNV) is a highly contagious virus endangering more than 120 species of marine and freshwater fish, which causes viral nervous necrosis (VNN), also known as viral encephalopathy and retinopathy (VER) [5]. The virus replicates in the central nervous system and retina of the affected fish, leading to neurological signs such as erratic swimming, blindness, and high mortality rates, particularly in larval and juvenile fish [6]. The NNV genome contains two single-stranded positive-sense RNAs (RNA1 and RNA2). Genomic RNA1 encodes RNA-dependent RNA polymerase (RdRp), responsible for viral genome replication, and RNA2 encodes the capsid protein (CP), which is the sole structural protein [7]. RGNNV is the most prevalent genotype worldwide, capable of infecting a wide range of teleost fish with a mortality rate of up to 100%, especially in various species of Perciformes [8]. In recent years, global warming also has posed a huge challenge to the aquatic environment. Scientists have investigated NNV extending its range into temperate regions from tropical and subtropical warmer waters, after already proliferating in environments previously unsuitable for its survival [9,10]. Thus, studying the virus–host interplay, and especially investigating viral infection on a multi-omics level, is crucial for elucidating the immune interactions and defense strategies of RGNNV in Tomato hind.

To date, multiple studies have focused on the impact of RGNNV on mRNA transcription in different tissues and cell lines of teleost fish [6,11,12,13,14,15]. Previous studies have shown that NNV infection significantly alters the transcriptional profile of host cells, inducing substantial changes in immune-related genes and pathways, which trigger inflammatory responses, apoptosis, and necrosis. In addition, a large number of microRNAs have been identified in RGNNV-infected *Epinephelus coioides* GF-1 cells, which are involved in regulating the interactions between RGNNV and the host [16]. However, the post-transcriptional regulatory mechanisms involved in RGNNV infection in Tomato hind remain unexplored.

In vertebrates, the non-coding RNA plays crucial roles in post-transcriptional regulation of genes and accounts for over 90% of the genomic sequences, although it constitutes less than 2% of the genome [17,18]. MicroRNAs (miRNAs), a class of 19–25 nucleotide non-coding RNAs, are among the most extensively characterized non-coding RNAs and play critical regulatory roles in essential biological processes such as cellular differentiation, organismal development, and immune responses [19,20]. As key regulators for activating or inhibiting immune responses, differently expressed miRNAs can either promote or inhibit pathogen replication by modulating the expression of target genes. Long non-coding RNAs (lncRNAs), longer than 200 nt, participate in virtually all biological processes within organisms, with particularly significant roles in immunological processes [21]. Fifteen circular RNAs (circRNAs) exhibit ubiquity and diversity across numerous species and play crucial regulatory roles in the pathogenesis and immune processes associated with various disease [22]. In recent years, whole transcriptome sequencing technology has been applied to multiple directions of research, including human diseases [23,24,25], environment stress, and the environmental toxicology response of aquatic organisms [26,27,28]. Among ncRNAs and mRNA in whole transcriptome profiles, the competing endogenous RNA (ceRNA) regulated gene expression by competitively binding miRNA [29]. Nevertheless, relevant information regarding ncRNAs and their regulatory mechanisms in the antiviral response during RGNNV infection in Tomato hind remains to be elucidated.

This research firstly investigated the potential functions of ncRNAs in Tomato hind infected with RGNNV utilizing whole-transcriptome sequencing. This study identified miRNAs, lncRNAs, and circRNAs linked to innate immune and cell death regulation to RGNNV infection, elucidating the underlying network and biological functions of key lncRNA–miRNA–mRNAs in performing diverse regulatory functions. This inquiry offers new theoretical viewpoints on the host–RGNNV molecular mechanism in groupers and provides valuable data for preventing and treating NNV-related diseases, aiming to aquatic organism safety protection.

## 2. Materials and Methods

### 2.1. Viral Infection and Collection of Fish Samples

We reared the *C. sonnerati* larvae at the coastal hatchery of Laizhou Mingbo Aquaculture Co., Ltd (Yantai, China). The hatchery had 32 culture pools with same quantity of fertilized eggs per tank. Each breeding pond was an indoor circular cement pond (volume, 40 m^3^; diameter, 6.6 m; water depth, 1 m). Water quality parameters were controlled as follows: temperature, 27.5 ± 1.0 °C; salinity, 29 ± 1 ppt; dissolved oxygen (DO), 5.5 ± 0.5 mg/L; pH, 8.0 ± 0.2; ammonia, 0.5 ± 0.5 ppm; and nitrite, 0.25 ± 0.25 ppm. Fry death due to natural viral infection began on day 20 of incubation, with clinical signs such as eyeball enlargement, skin darkening, reduced swimming ability, and surface floating.

Owing to the small size of the fry, whole fish were used for nucleic acid extraction and NNV detection via polymerase chain reaction (PCR). Using the RNA simple Total RNA Kit (TIANGEN, Beijing, China), we isolated RNA from 30 infected fry from three disease seedling pools and 30 healthy fry from three normal seedling pools, respectively. Next, cDNA was synthesized from the RNA samples according to HiScript III RT SuperMix for qPCR (Vazyme, Nanjing, China). The quality and quantity of DNA and RNA samples were evaluated by measuring absorbance at OD 260 and 280 nm with a NanoDrop spectrophotometer (Thermo Fisher Scientific, Waltham, MA, USA). NNV and RSIV from the obtained cDNA samples were diagnosed using PCR technology. The PCR primers of NNV and RSIV used in this study were as shown in Table S1. The virus detection results were as shown in Figure S1. The RGNNV and RSIV cDNA samples previously isolated from grouper were used as the positive control, and the healthy sample with no virus infection was used as the negative control.

Sixty healthy brain tissues and 60 infected brain tissues were sampled at −80 °C for sequencing analysis. Each sample consisted of brain tissue from 20 fry, with three replicates prepared for the infected group (NNV-1, NNV-2, NNV-3) and the control group (Control-1, Control-2, Control-3). Subsequently, the samples were promptly preserved in liquid nitrogen and kept at a temperature of −80 °C until RNA extraction and sequencing.

### 2.2. Transcriptome Sequencing and Analysis

#### 2.2.1. Extraction of RNA Followed by Illumina Sequencing

Six brain samples selected from *C. sonnerati* fry from the control group and NNV-infected group were used for RNA isolation. Each group had three independent biological replications with 20 fry per replication. The concentration, quality, and integrity of RNA were detected using a NanoDrop spectrophotometer (Thermo Fisher Scientific, Waltham, MA, USA) after isolating total RNA by Trizol Reagent (Thermo Fisher Scientific, Waltham, MA, USA).

#### 2.2.2. Library Construction and Sequencing of miRNA, lncRNA and circRNA

Six miRNA libraries were performed using the NEBNext Multiplex Small RNA Library Prep Set for Illumina (New England Biolabs, Inc., Ipswich, MA, USA) on the Illumina Novaseq X Plus (Illumina, Inc., San Diego, CA, USA). We obtained clean data via removing the adapter and low-quality sequences. Unique reads with 18–25 nt were performed by BLAST analysis using the miR Base 22.0 database (http://microrna.sanger.ac.uk., accessed on 16 January 2026) to identify and annotate miRNAs.

Six lncRNA libraries were constructed according to Illumina NovaSeq X Plus (Illumina, USA), Illumina NEBNext Ultra RNA Library Prep Kit (New England Biolabs, Inc., Ipswich, MA, USA), and paired-end sequencing was performed on an Illumina Novaseq X plus (Illumina, USA). Raw data were filtered to obtain high quality reads (i.e., clean reads) to ensure data reliability. Clean reads were subsequently aligned to the reference genome with HISAT2 (v2.1.0). LncRNA identification was based on: (i) transcript reconstruction using Stringtie software (v 2.2.1), (ii) transcript length ≥200 base pairs (bp) and exons ≥2, (iii) selection of anti-sense, intergenic nd intronic lncRNAs, (iv) lncRNAs with coverage > 3 in at least one sample, and (v) transcript coding ability, as predicted by PLEK (version 2.0), CNCI (Version 1.0) and Pfamscan (version 1.6.4).

For circRNA libraries, HISAT2 (version 2.1.0) was used to blast reads to the reference genome. We intercept 20 bp in two ends of unmapped reads as the anchor reads using Tophat2 (version 2.0.14). Then, we align these reads to the genome again for circRNA detection using Bowtie2 (v2.5.1). find_circ (version 1.0) was used to identify circRNAs, the criteria for which were breakpoints = 1, anchor_overlap ≤ 2, edit ≤2, and the length <100 kb. Then, we identified the parental genes of the circRNAs based on their genomic location annotation information.

### 2.3. Analysis of Expressed ncRNAs and Functional Enrichment

To identify DE miRNAs between the control group and NNV-infected group, the expression levels of miRNAs were also normalized, the screened condition for which was |log2FoldChange| > 1, significance *p* < 0.05. Target genes of DE miRNAs were predicted using MiRanda (version 3.3a) and performed using the function analysis of GO and KEGG enrichment (*p* < 0.05).

We used Fragments Per Kilobase of transcript per Million mapped reads (FPKM) to standardize the expression of lncRNA. DE lncRNAs were identified with the screening threshold set to |log2(fold change)| > 1 and a *p* < 0.05. To predict the function of DE lncRNAs, cis-and trans lncRNA were used to predict target genes to perform GO and KEGG enrichment analyses.

We quantified circRNAs expression using the transcripts per kilobase of exon model per million mapped reads (TPM). DESeq (v1.38.3) was applied to screen for DE circRNAs, with significance set at |log_2_FC| > 1 and *p* < 0.05. GO and KEGG enrichment analyses of the source genes of these DE circRNAs were then analyzed for GO and KEGG enrichment analyses.

### 2.4. lncRNA–miRN–AmRNA Regulatory Network Construction

We selected targeted DE mRNAs of DE miRNAs from the top 20 KEGG pathways with |log2(fold change)| > 2 and FDR < 0.05. Then, we generated protein–protein interaction (PPI) networks among these selected targeted genes through Search Tool for the Retrieval of Interacting Genes (STRING) database (http://string-db.org/, accessed on 6 February 2026). The interactions were also analyzed in the STRING database with high confidence (interaction score > 0.500) and visualized in Cytoscape software (v3.10.4).

Then, we used miRNA–mRNA and miRNA–lncRNA interaction pairs to predict the associated ceRNA regulatory network. The predicted regulatory relationship was required to meet the following criteria: a negative correlation *p* < 0.05 and a Spearman’s correlation coefficient <−0.8 based on mRNA, miRNA, and lncRNA expression levels. Finally, a ceRNA regulatory network of lncRNA–miRNA–mRNA was constructed following *p* < 0.05 and Spearman’s correlation coefficient <−0.5.

### 2.5. qPCR Experimental Validation

We synthesized cDNA from the extracted total RNA using the HiScript III RT SuperMix for qPCR (+gDNA wiper) (Nanjing Vazyme Biotech Co., Nanjing, China), according to the manufacturer’s protocol. The expression patterns of 5 DE miRNAs, 5 DE lncRNAs, and 5 DE circRNAs were examined by qRT-PCR, randomly. qPCR primers were designed using Primer 5.0 (Supplementary Table S1) and the reactions were prepared with Trans Gen’s Trans Start Top Green qPCR SuperMix (Beijing TransGen Biotech Co., Beijing, China) on a Step One Plus Real-Time PCR System (Applied Biosystems, Foster, CA, USA) according to the manufacturer’s protocol. The expression levels of 20 RNAs were evaluated using the 2− ΔΔCt comparative Ct method [30].

## 3. Results

### 3.1. Transcriptome Sequencing Data of miRNAs, lncRNAs, circRNAs

**Figure 1:**
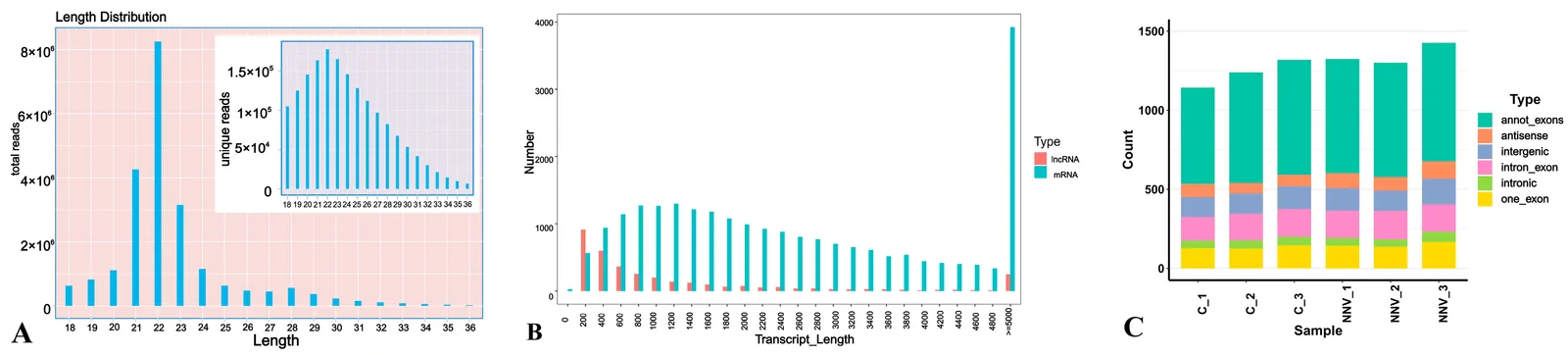
Main length distribution of clean reads in six libraries. (**A**) miRNAs length distribution of clean reads. (**B**) lncRNAs length distribution of clean reads. (**C**) cirRNAs type distribution of clean reads.

In total, 144.53 million raw reads were acquired, which underwent quality control processing resulting in 126.23 million clean reads in six miRNA libraries (Supplementary Table S1). The mapping results showed that average 72.01% of these clean reads mapped to the reference genome sequence, of which Q20 ≥ 99.29%, Q30 ≥ 97.09%. The predominant length distribution of the clean reads is illustrated in Figure 1A. A total of 2357 miRNAs were identified, among which 392 were new miRNAs.

In the statistics of lncRNA and mRNA libraries, 114,102,156, 12,3769,322, 128,412,256, 147,236,846, 14,0748,970, and 174,783,932 raw reads from the C-1, C-2, C-3, NNV-1, NNV-2, and NNV-3 groups were respectively mapped to the reference genome (Supplementary Table S2). A total of 98.61–99.04% of these clean reads were mapped to the reference genome sequence (Supplementary Table S3). Meanwhile, according to the data reflecting the quality of sequencing, such as Q20 ≥ 98.08%, Q30 ≥ 94.68%, and GC content ≈50%, all the quality of the sequencing data of six libraries (lncRNA and small RNA, respectively) was in good condition, which could ensure the accuracy of subsequent analysis and research (Supplementary Table S3). Up to 67.18% of the lncRNAs were 200–1000 bp, while 7.27% were more than 5000 bp (Figure 1B). A total of 2830 lncRNAs and 3480 lncRNA transcripts were identified.

In the statistics of circRNA libraries, 222.22 million reads were mapped to the reference genome sequence (Supplementary Table S4). The map percentage reached up to 84.69%. According to the generation regions of circRNAs in the genome, these circRNAs were classified into six types: exon, intron_exon, intronic, antisense, intergenic circRNAs, and the annot_ exons circRNAs accounted for the largest percentage (Figure 1C). A total of 2749 circRNAs were identified.

### 3.2. Analysis of Differentially Expressed miRNAs, lncRNAs, circRNAs

**Figure 2:**
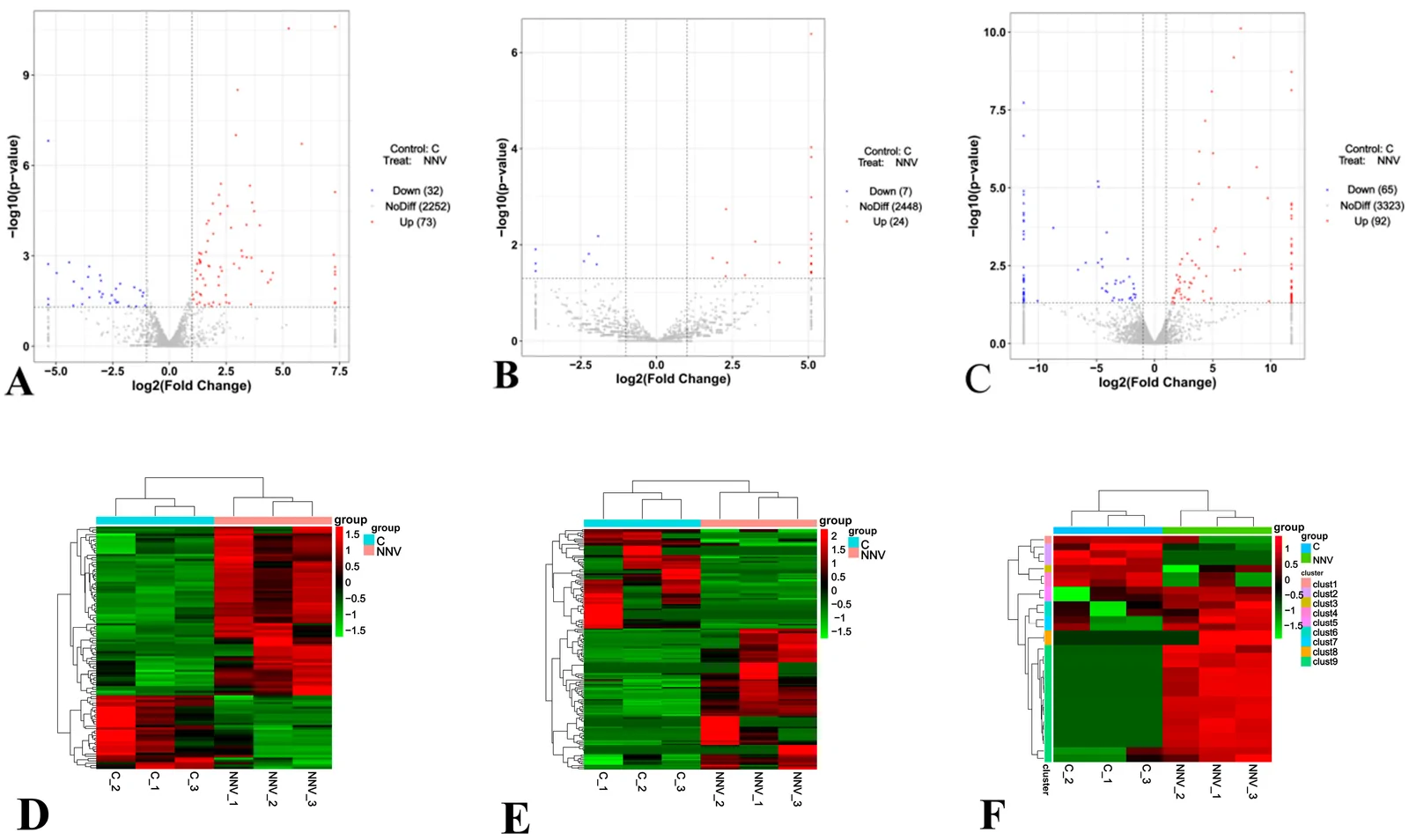
DE miRNAs, DE lncRNAs, DE circRNAs of brain in *C. sonnerati* infected with RGNNV. (**A**) The volcano plots of DE miRNAs. (**B**) The volcano plots of significantly expressed lncRNAs. (**C**) The volcano plots of DE circRNAs. (**D**) The heatmaps of DE miRNAs. (**E**) The heatmaps of DE lncRNAs. (**F**) The heatmaps of DE circRNAs. The significance threshold was set to *p* < 0.05, *n* = 3.

A total of 157 DE lncRNAs, 105 DE miRNAs, and 31 DE circRNAs were significantly altered in *C. sonnerati* brain (Figure 2A–F). To illustrate the relationship between fold change and *p*-value, we generated volcano plot and heatmap in Figure 2. These findings demonstrate distinct expression patterns of differentially expressed ncRNAs following RGNNV infection.

### 3.3. Functional Enrichment Analysis of Differentially Expressed RNA Transcripts

#### 3.3.1. GO Enrichment Analysis of Differentially Expressed Transcripts

**Figure 3:**
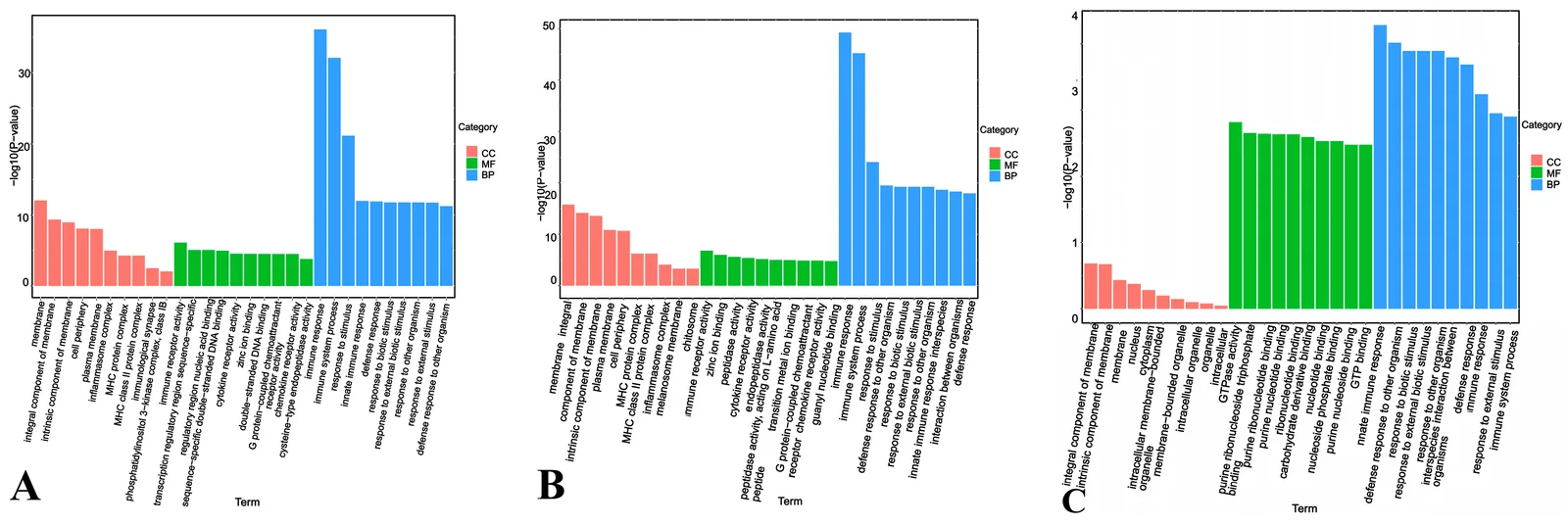
GO enrichment of DE miRNAs, DE lncRNAs, DE circRNAs targeted genes in brain. (**A**) The GO enrichment of DE miRNAs targeted genes. (**B**) The GO enrichment of DE lncRNAs targeted genes. (**C**) The GO enrichment of DE circRNAs targeted genes. The line graph is the number of genes belonging to the corresponding GO term and the bar graph is taken from log base 2 of the *p*-value. Different *p* values are shown in different colors. The significance threshold was set to *p* < 0.05, *n* = 3.

**Figure 4:**
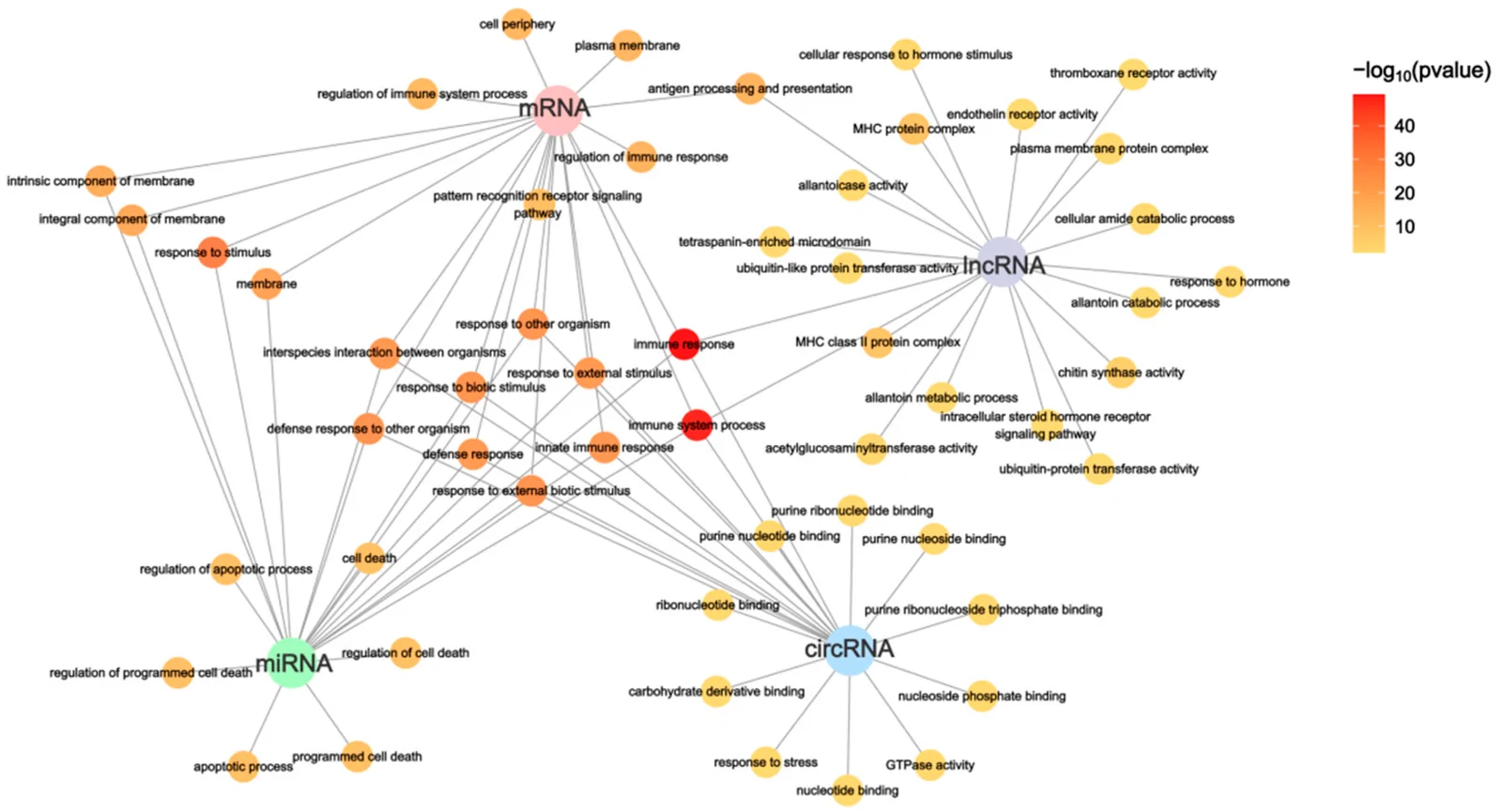
GO enrichment of DE lncRNAs, DE miRNAs, DE circRNAs, DE mRNAs in brain. The line graph is the number of genes belonging to the corresponding GO term and the bar graph is taken from log base 2 of the *p*-value. Different *p* value are shown in different colors. The significance threshold was set to *p* < 0.05, *n* = 3.

In order to further understand the regulatory functions, we perform GO and KEGG enrichment analysis of DE miRNAs, DE lncRNAs, and DE circRNAs targeted genes (Figure 3A–C). GO terms analysis the top three enriched terms under CC were “membrane”, “integral component of membrane”, and “intrinsic component of membrane” (*p* < 0.05). The top three enriched terms under BP were “immune response”, “immune system process”, and “response to stimulus” (*p* < 0.05), and the top three enriched terms under MF were “immune receptor activity”, “transcription regulatory region sequence-specific DNA binding”, and “regulatory region nucleic acid binding”. Remarkably, the biological process occupies the highest proportion. This indicated that complex biological processes occur in Tomato hind during viral infection. Interestingly, the GO terms of DE lncRNAs were similar to DE miRNAs, suggesting that miRNAs could interact with lncRNAs during the immune response process. For circRNAs GO analysis, the top three enriched terms under MF were “anion binding”, “ion binding”, and “carbohydrate derivative binding” (*p* < 0.05), and the top three enriched terms under BP were “innate immune response”, “response to external stimulus”, and “regulation of synaptic transmission” (*p* < 0.05). DE miRNAs, DE lncRNAs, DE circRNAs, and DE mRNAs were involved in many of the same immune related process, such as innate immune response, immune process, and defense response (Figure 4).

#### 3.3.2. KEGG Enrichment Analysis of Differentially Expressed Transcripts

**Figure 5:**
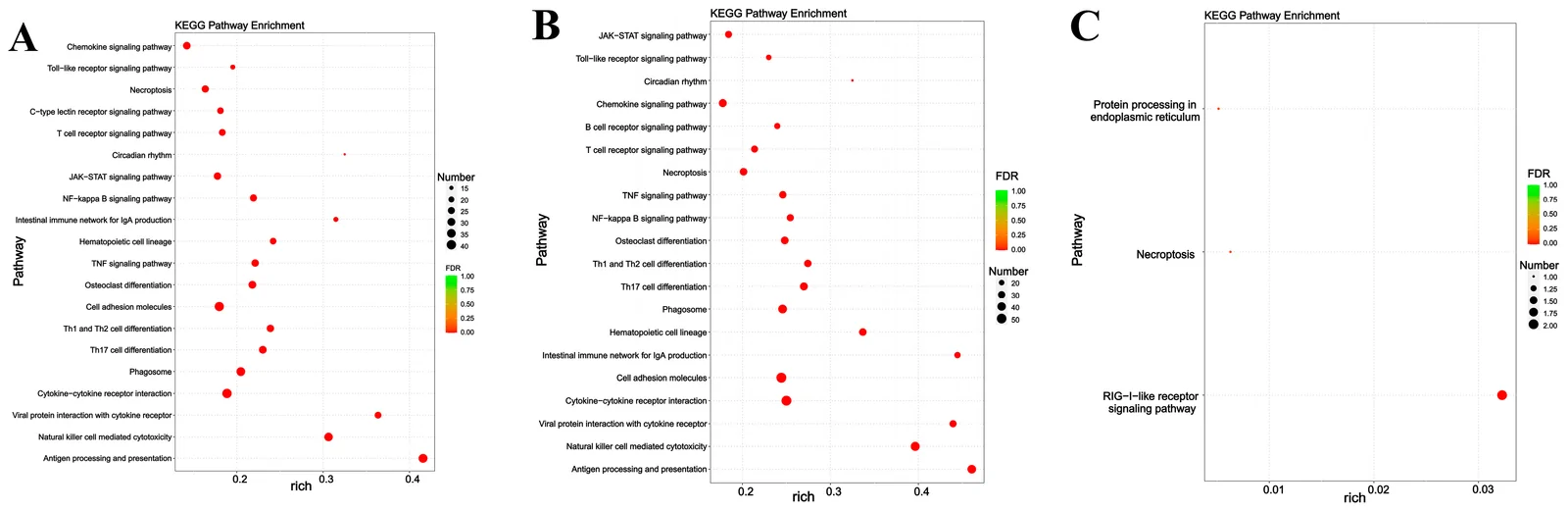
Enrichment KEGG pathways of DE miRNAs, DE lncRNAs, and DE circRNAs in brain. (**A**) KEGG enrichment of DE miRNAs. (**B**) KEGG enrichment of DE lncRNAs. (**C**) KEGG enrichment of DE circRNAs. The top 20 enrichment pathways are shown in the rainbow bubble chart. “Rich factor” means that the ratio of the DE RNAs number and the number of genes has been annotated in this pathway. The size of the circle represents the number of genes and the color represents different *p*-value. The significance threshold was set to *p* < 0.05, *n* = 3.

**Figure 6:**
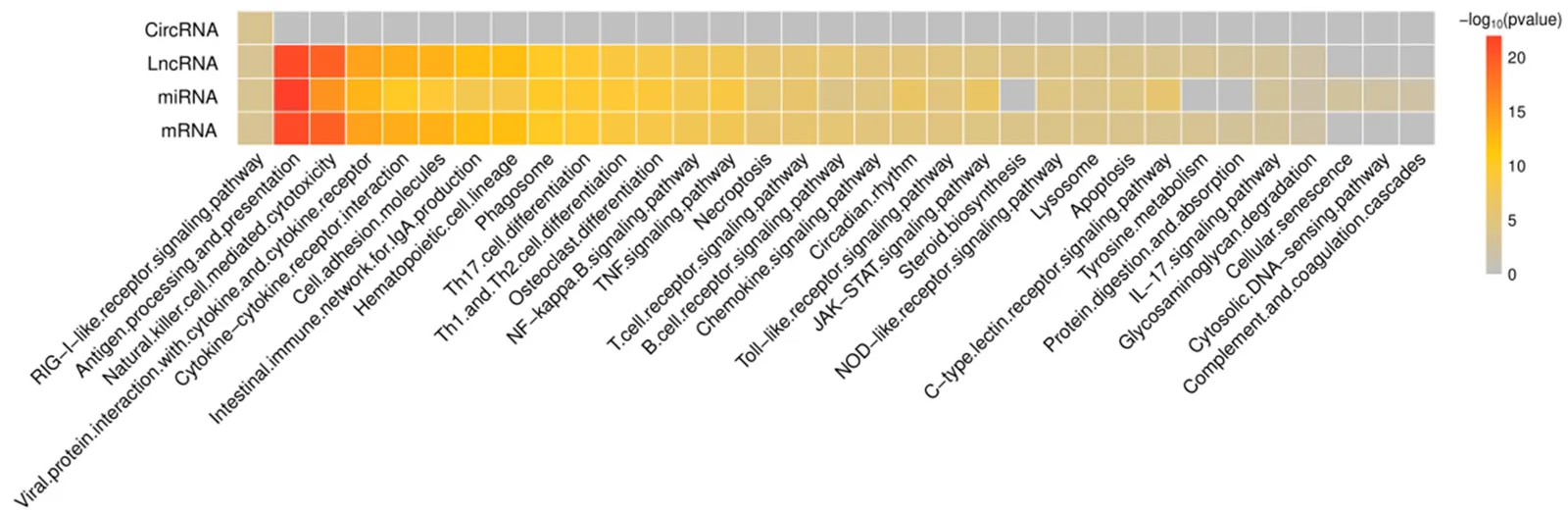
Common enrichment KEGG pathways of four kinds of DE RNAs in brain. Top 30 KEGG enrichment pathways of ≥2 kinds of RNA. Different log base 2 of the *p* value are shown in different colors. The significance threshold was set to *p* < 0.05, *n* = 3.

The DE ncRNAs that were infected by RGNNV were further classified by their function in the Kyoto Genes and Genomes (KEGG) pathway. As shown in Figure 5A, DE miRNAs in the brain were mainly involved in antigen processing and presentation, viral protein interaction with cytokine and cytokine receptor, cell adhesion molecules, phagosome, NF-kappa B signaling pathway, TNF signaling pathway, necroptosis, apoptosis, JAK-STAT signaling pathway, chemokine signaling pathway, and multiple PPAR pathways. The KEGG pathways of DE lncRNAs were extremely consistent with DE miRNAs (Figure 5B). The KEGG pathway of DE circRNAs is only “RIG-1 like receptor signaling pathways” (Figure 5C). Furthermore, this study summarized the top 30 pathways of four kinds of RNAs that were enriched in brain (Figure 6). The results showed that DE miRNAs, DE lncRNAs, and DE mRNAs in the brain were all involved in innate immune response and cell death related pathways (Figure 6).

### 3.4. Construction of lncRNA–miRNA–mRNA Regulatory Networks

**Figure 7:**
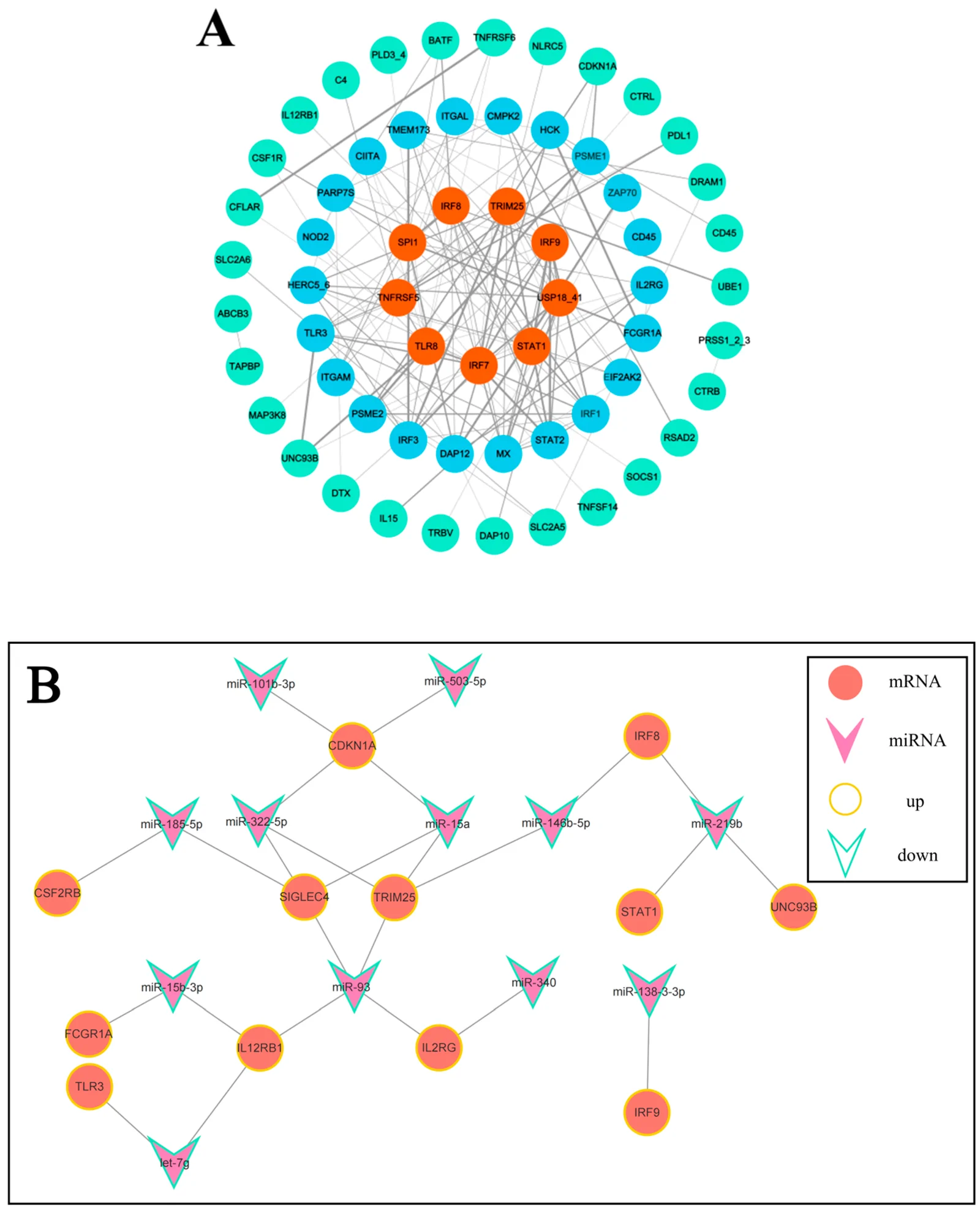

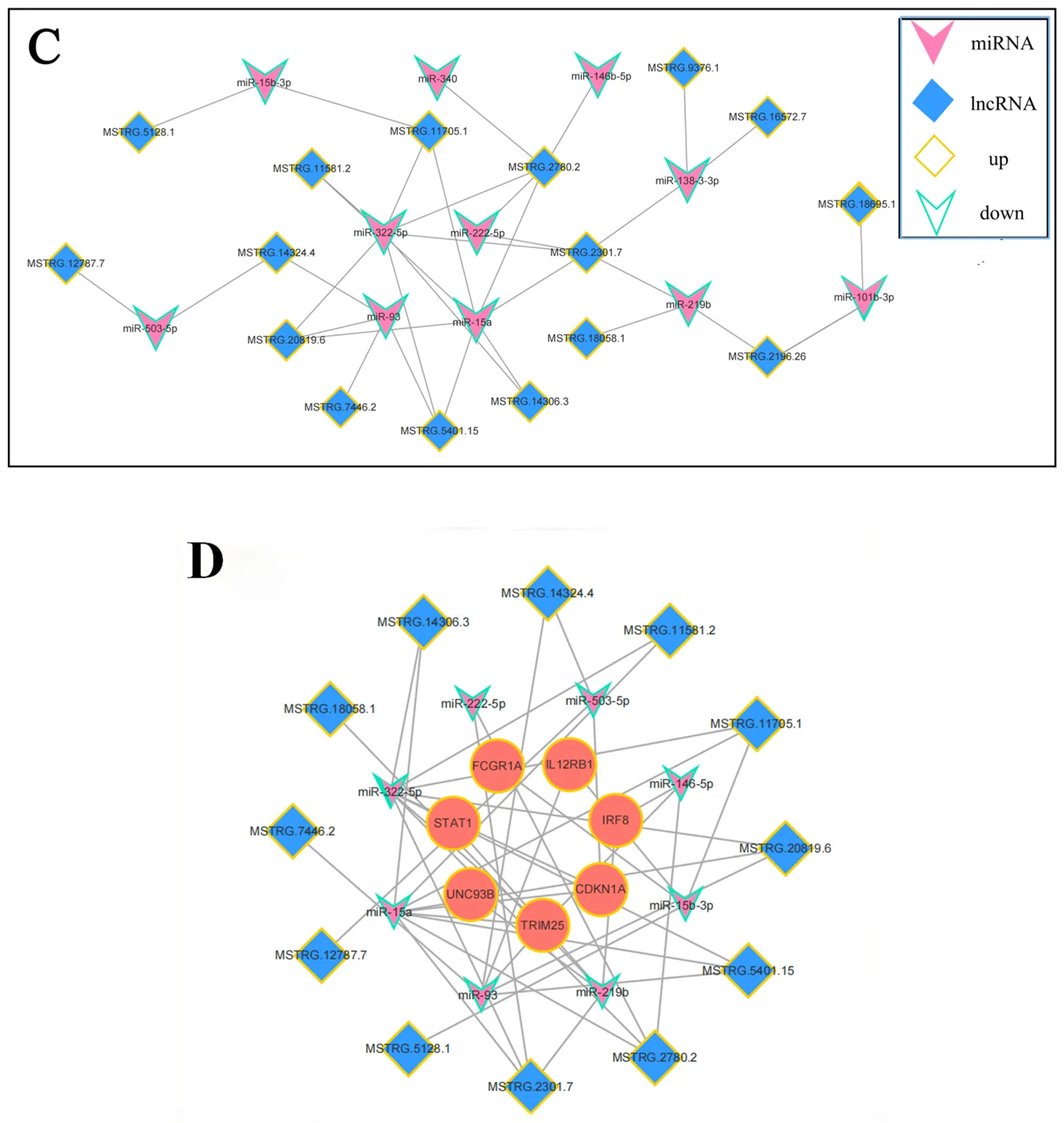
lncRNA–miRNA–mRNA regulatory network in the control and NNV-infected groups. (**A**) PPI network based on targeted DE mRNAs of DE miRNAs from the top 20 KEGG pathways with |log2(fold change)| > 2 and FDR < 0.05. Note: Orange, blue, and green circles represent interaction scores from high to low, respectively. (**B**) mRNA–miRNA network. (**C**) lncRNA–miRNA network. (**D**) lncRNA–miRNA–mRNA network. Note: Red circles, pink arrows, blue rhombus represent DE mRNAs, DE miRNAs, and DE lncRNAs, respectively. Yellow and green outlines denote positive and negative regulation, respectively.

The PPI network of DE mRNAs targeted by DE miRNAs were predicted using the STRING database in the top 20 KEGG pathways (Figure 7A). The expression patterns of all identified DE mRNAs are mapped and highlighted in the *C. sonnerati* genome (Figure 7A). The results demonstrated that many immune-related regulatory genes, pro-inflammatory factor genes, and ubiquitin proteasome system genes were highly enriched in the NNV-infected brain. These key DE mRNAs were three times more up-regulated in the NNV-infected group than in the control group. Moreover, the DE mRNAs with degree ≥10 in the NNV-infected group included *IRF7, IL10, STAT1, USP18_41, IRF9, IRF8, TLR8, TNFRSF5, TRIM25, SPI1, IRF1, STAT2, TLR3, MX,* and *IRF3* (Figure 7A).

Subsequently, we constructed lncRNA–miRNA–mRNA networks to further explore how ncRNA regulates the immune response to RGNNV in brain. In brief, we generated the miRNA–mRNA pairs (Figure 7B) and miRNA–lncRNA pairs (Figure 7C) based on the principle of negative regulation in the top 20 KEGG pathways. Then, we constructed a ceRNA regulatory network of mRNA–miRNA–lncRNA with negative Spearman’s correlation coefficient (<−0.8), which responded to NNV infection in *C. sonnerati* brain. In the control vs NNV-infected comparison, seven DE mRNAs, nine DE miRNAs, and 13 DE lncRNAs formed a ceRNA regulatory network (Figure 7D). *STAT1, IRF8, TRIM25, IL12RB1, CDKN1A, FCGR1A,* and *UNC93B* were regulated by one or multiple miRNA and lncRNA. ncRNA were involved in immune-related signaling pathway via regulating the expression of *UNC93B, IRF8, STAT1, IL12RB1,* and *TRIM25*, including JAK-STAT signaling pathway, NF-kB signaling pathway, and PPAR signaling pathway. ncRNAs may regulate the expression of *FCGR1A* and *CDKN1A* to promote the process of cell death.

### 3.5. Validation of DE mRNA, DE lncRNA, DE miRNA, and DE circRNA by qRT-PCR

**Figure 8:**
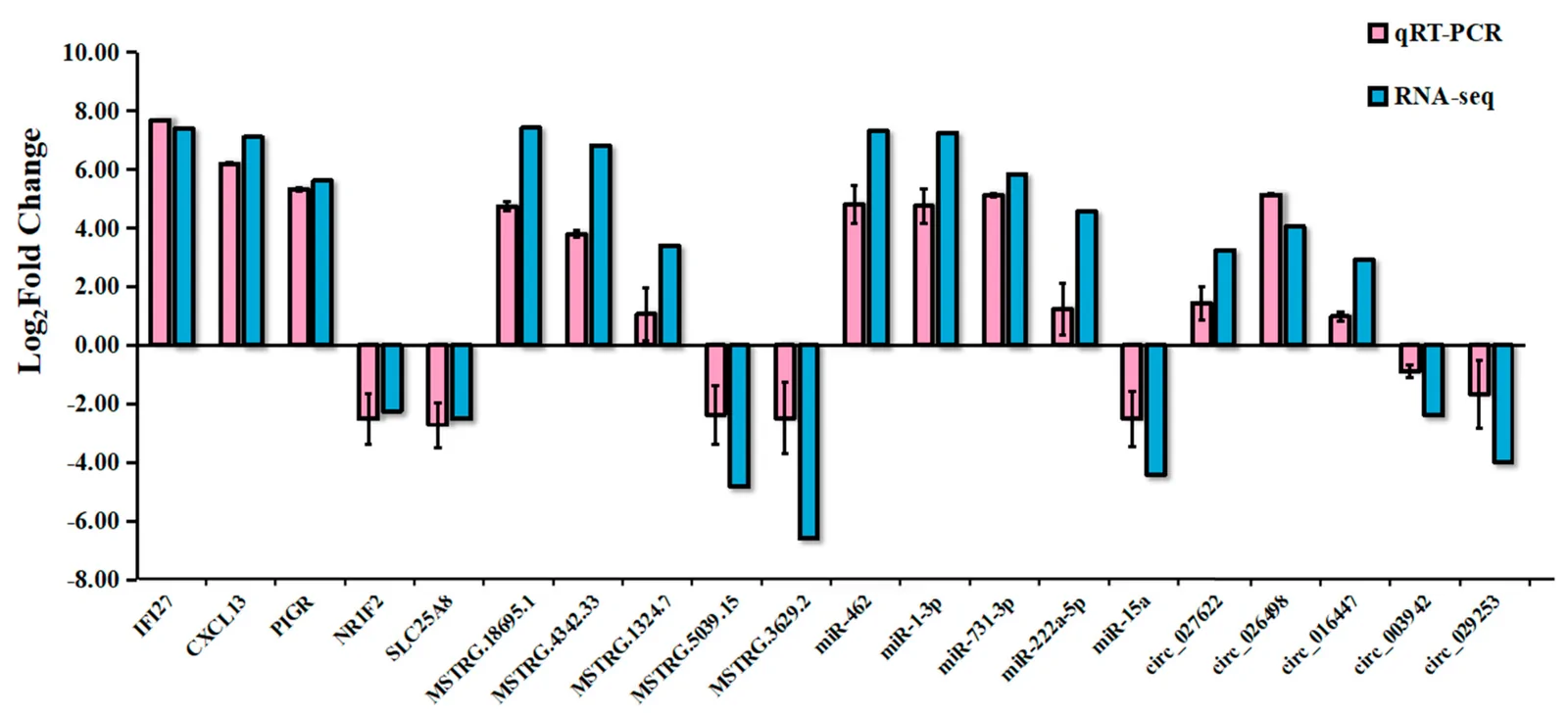
qRT-PCR verification of randomly selected five DE lncRNAs, five DE miRNAs, five DE circRNAs, and five targeted DE mRNAs. Each vertical bar represents the mean ± SD (*n* = 3).

DE miRNAs, DE lncRNAs, DE circRNAs, and DE mRNAs in the control group and NNV-infected group were quantified by using qRT-PCR. The relative expression (|log2(fold change)|) of these DE RNAs was similar between qPCR and RNA sequencing data, although some mini differences were observed (Figure 8). The RNA-seq results indicated that our RNA sequencing results were reliable and suitable for bioinformatics analysis.

## 4. Discussion

Viral infections have long severely restricted the survival of wild fish populations and the development of aquaculture. RGNNV posed a significant challenge to grouper aquaculture, leading to considerable economic losses. In our study, a natural RGNNV outbreak severely decimated the seedling population during artificial breeding of *C. sonnerati*. Recent studies highlighted the potential of ncRNAs as fresh biomarkers for accurate disease diagnosis and monitoring treatment outcomes, which is essential for managing and preventing disease [31,32,33]. The brain, as the central nervous system of fish, is the core invasive organ of RGNNV. In the current study, we constructed ncRNA transcriptomic libraries to reveal the molecular regulatory landscape in *C. sonnerati* brain during RGNNV infection.

Our sequencing analysis showed that ncRNAs exhibited significant differential expression following NNV infection, with significant enrichment in multiple immune-related and cell death-related biological processes or signaling pathways. GO analysis suggested that immune response processes predominantly take place in the cell membrane surface, and relied on immune receptors to counteract viral invasion. KEGG analysis revealed that RGNNV infection triggered multiple signaling cascades in the brain tissue of *C. sonnerati*. Notably, the high activation of the TNF signaling pathway, apoptosis and necroptosis signaling pathway may lead to neuron necrosis in fish brain, which regulated cell death transduction in this study [34,35]. TNF can not only directly induce the expression of inflammatory genes, but also indirectly promote inflammation by inducing cell death, which were all associated with the phenotype, specifically necroptosis. Moreover, similar pathways were enriched in the process of other fishes responding to NNV infection [11,12,13,14]. These pathways suggested that aberrant activation of apoptotic programs may be one of the key factors contributing to the drastic reduction in larval population numbers during RGNNV infection.

In addition, the enrichment of the PRR signaling pathway, JAK-STAT signaling pathway, and cell adhesion molecule signaling pathway reflected the host’s immune defense attempt to eliminate the virus through the interferon system. PRRs play a critical role in recognizing specific patterns of microbial components, eliminating antigens and activating innate immunity [36,37]. The interferon (IFN) system serves as a core component of innate immunity against viral infections, which is essential for regulating the transcription of hundreds of antiviral genes via the JAK-STAT signaling pathway [38]. The significant enrichment of cell adhesion molecules also indicated intense communication among brain cells. Cell adhesion is essential in immunity, which participates in the cellular immune responses of encapsulation and nodule formation [39]. Phagosomes and phagolysosomes play a very important role in the immune system by removing foreign substances [40]. Studies have reported that similar immune signaling pathways were involved in zebra fish larvae, and in spleen and liver of *Micropterus salmoides* with LMBV infection [41,42]. Our team also conducted the transcriptome sequencing of artificial NNV-infected TGB cells within 12 h post-infection [43]. In the TGB cell model artificially infected with RGNNV, the enriched immune-related pathways were largely consistent with those of ncRNA target genes in our study, suggesting that these ncRNAs may be involved in antiviral responses and pathology via regulating specific mRNAs in *C. sonnerati* brain.

Numerous research studies have clarified that the complicated regulatory network created by lncRNA–miRNA–mRNA has a vital function in regulating various immune pathways [44,45,46,47]. From common enrichment GO and KEGG pathways, we inferred that miRNA and lncRNA may participate in the immune response in the brain of Tomato hind via interacting with mRNA. To understand further insights into the interactive landscape between Tomato hind and RGNNV, we constructed an interaction network, specifically reflecting cell death and innate immune-associated signaling pathways based on the PPI network. Furthermore, we predicted a lncRNA–miRNA–mRNA regulatory interaction network. We observed that a single miRNA could be competitively sequestered by multiple lncRNAs, thereby modulating the expression of target genes enriched in immune-related pathways.

In our ceRNA network, miR-93 exhibited the higher node connectivity, indicating a potential core role in the immune response. It is reported that the brain vacuolation of fishes caused by RGNNV infection is generally attributed to aberrant inflammatory responses and neurons cell death [6,12,48,49]. In mice, miR-93 could effectively inhibit traumatic brain injury (TBI) induced inflammatory responses by promoting the polarization of M2 macrophages, thereby reforming the damaged micro-environment and promoting brain tissue repair [50]. Other research in the mouse model proved that neurons and oligodendrocytes are the targeted cells of the IL-12 signaling pathway. Targeted knockout of the IL-12 receptor gene (IL12RB2) could restore myelin homeostasis and neuronal function [51]. As a result, we speculated that increasing levels of MSTRG.14324.4, MSTRG.20819.6, MSTRG.5401.15, and MSTRG.7446.2 may potentially increase IL12RB1 levels via binding miR-93 to promote inflammation in brain neurons, leading to *C. sonnerati* brain injury. These findings implied that the lncRNA–miRNA–mRNA regulatory network in *C. sonnerati* brain may act as a key immune regulatory hub, and its disruption may potentially aggravate viral-induced neuroinflammation and cell death.

Additionally, some lncRNAs were predicted to promote *TRIM25, STAT1*, and *UNC93B* expression by sponging miRNA, suggesting that the ceRNA network not only participated in pathological damage but also mediated a limited innate antiviral defense. TRIM25 not only binds to viral proteins to reduce viral replication, but also participates in the RIG-I signaling pathway to induce IFN-1 production and activate innate antiviral immune responses [52]. As a core transcription factor in the IFN-I pathway, the stability and activity of *STAT1* directly regulate the efficacy of the antiviral immune response [53]. *UNC93B* is a critical molecule required for *TLR3* to recognize double-stranded RNA viruses and to achieve precise localization and signal transduction [54]. Interestingly, miR-93 was predicted to up-regulate *TRIM25* by the competition of lncRNAs in our ceRNA network. Collectively, these results suggested that miR-93 may serve as promising therapy targets for combating RGNNV infection.

However, a limitation of this study is that we only performed transcriptomic analysis and quantitative validation to confirm the reliability of the omics data, but did not conduct functional validation. In particular, the construction of the lncRNA–miRNA–mRNA regulatory network was based on predictive analyses of potential molecular interactions, and therefore cannot be directly regarded as functional evidence.

Future studies should prioritize the molecular validation of the regulatory functions of the candidate genes identified in this study, including RNA interference (RNAi) and over-expression assays both in vivo and in vitro levels. In addition, dual-luciferase reporter assay is indispensable for validating these predicted regulatory axes. Furthermore, miR-93 was identified as a potential modulator involved in neuroinflammation and antiviral immunity, making it a promising target for further studies and highlighting its potential role in host immune defense in *C. sonnerati*.

## 5. Conclusions

This study provides the first ncRNA transcriptome data of *C. sonnerati* infected with RGNNV, identifies key genes and pathways involved in RGNNV infection, and constructs a lncRNA–miRNA–mRNA network, thereby establishing a theoretical basis for elucidating the potential immune response mechanism in this species. Taken together, these results offer a potential molecular regulatory landscape of immune response in *C. sonnerati* and provide a significant foundation for future functional studies in groupers.

## Data Availability

The raw data was submitted to the NCBI database (SRA database PRJNA1293526). The Supporting Materials are published as submitted, without typesetting or editing. The responsibility for scientific accuracy and content remains entirely with the authors.

## Supplementary Materials

The following supporting information can be downloaded at: https://www.mdpi.com/article/10.3390/biology15161379/s1, Figure S1: The virus detection in *C. sonnerati* brain tissue by PCR. Table S1: Sequences of primers used in experiment. Table S2: Data statistics of miRNA sequencing. Table S3: Data statistics of lncRNA sequencing. Table S4: Statistics after lncRNA data filtering. Table S5: Data statistics of circRNA sequencing.

## References

[1] Shpigel M., Fishelson L. (2010). Territoriality and associated behaviour in three species of the genus *Cephalopholis* (Pisces, Serranidae) in the gulf of aqaba, red sea. J. Fish Biol..

[2] Shpigel M., Fishelson L. (1989). Food habits and prey selection of three species of groupers from the genus *Cephalopholis* (Serranidae, Teleostei). Environ. Biol. Fish.

[3] Shpigel M., Fishelson L. (1989). Habitat partitioning between species of the genus *Cephalopholis* (Pisces, Serranidae) across the fringing reef of the Gulf of Aqaba (Red Sea). Mar. Ecol. Prog. Ser..

[4] Toffan A., Marsella A., Menconi V., Bertola M. (2025). Finfish infectious diseases in the Mediterranean basin: A systematic review with insights on vaccination possibilities. Fish Shellfish Immunol..

[5] Jungi S.V., Sangsuriya P., Taengphu S., Phiwsaiya K., Sonthi M., Nuangsaeng B., Salin K.R., Senapin S., Dong H.T. (2022). Detection of nervous necrosis virus RGNNV genotype in pearl gentian grouper (*Epinephelus lanceolatus* ♂ × *E. fuscoguttatus* ♀) fry imported to Thailand. Aquaculture.

[6] Wang Q., Peng C., Yang M., Huang F., Duan X., Wang S., Cheng H., Yang H., Zhao H., Qin Q. (2021). Single-cell RNA-seq landscape midbrain cell responses to red spotted grouper nervous necrosis virus infection. PLoS Pathog..

[7] Iwamoto T., Okinaka Y., Mise K., Mori K.I., Arimoto M., Okuno T. (2004). Identification of host-specificity determinants in betanodaviruses by using reassortants between striped Jack nervous necrosis virus and seven band grouper nervous necrosis virus. J. Virol..

[8] Shetty M., Shivakumar Santhosh K., Venugopal M.N., Karunasagar I. (2012). Betanodavirus of marine and freshwater fish: Distribution, genomic organization, diagnosis and control measures. Indian J. Virol..

[9] Thorstad E.B., Bliss D., Breau C. (2021). Atlantic salmon in rapidly changing environment-Facing the challenges of reduced marine survival and climate change. Aquat. Conserv. Mar. Freshw. Ecosyst..

[10] Cohen-Rengifo M., Danion M., González A., Laure Bégout M., Cormier A., Noel C., Cabon J., Vitré T., Mark F., Mazurais D. (2022). The extensive transgenerational transcriptomic effects of ocean acidification on the olfactory epithelium of a marine fish are associated with a better viral resistance. BMC Genom..

[11] Liu P., Wang L., Kwang J., Yue G.H., Wong S. (2016). Transcriptome analysis of genes responding to NNV infection in Asian seabass epithelial cells. Fish Shellfish Immunol..

[12] Chen W.J., Yi L.-Z., Feng S.S., Liu X.D., Asim M., Zhou Y.C., Lan J.F., Jiang S.J., Tu J.G., Lin L. (2017). Transcriptomic profiles of striped snakehead fish cells (SSN-1) infected with red-spotted grouper nervous necrosis virus (RGNNV) with an emphasis on apoptosis pathway. Fish Shellfish Immunol..

[13] Wang L.N., Tian Y., Cheng M., Li Z., Li S., Wu Y., Zhang J., Ma W., Li W., Pang Z. (2019). Transcriptome comparative analysis of immune tissues from asymptomatic and diseased *Epinephelus moara* naturally infected with nervous necrosis virus. Fish Shellfish Immunol..

[14] Tso C.H., Li M.W. (2018). Transcriptome profiling analysis of grouper during nervous necrosis virus persistent infection. Fish Shellfish Immunol..

[15] Peruzza L., Pascoli F., Dalla Rovere G., Franch R., Ferraresso S., Babbucci M., Biasini L., Abbadi M., Panzarin V., Toffan A. (2021). Transcriptome analysis reveals a complex response to the RGNNV/SJNNV reassortant Nervous Necrosis Virus strain in sea bream larvae. Fish Shellfish Immunol..

[16] Chen W.J., Yi L.-Z., Feng S.S., Liu X.D., Asim M., Zhou Y.C., Lan J.F., Jiang S.J., Tu J.G., Lin L. (2017). Characterization of microRNAs in orange-spotted grouper (*Epinephelus coioides*) fin cells upon red-spotted grouper nervous necrosis virus infection. Fish Shellfish Immunol..

[17] Rincon-Riveros A., Morales D.F., Rodríguez J.A., Villegas V.E., López-Kleine L. (2021). Bioinformatic tools for the analysis and prediction of ncRNA interactions. Int. J. Mol. Sci..

[18] Chen L.L., Narry Kim V. (2024). Small and long non-coding RNAS: Past, present, and future. Cell.

[19] Deveale B., Swindlehurst-Chan J., Blelloch R. (2021). The roles of microRNAs in mouse development. Nat. Rev. Genet..

[20] Vasconcellos R., Alvarenga É.C., Parreira R.C., Lima S.S., Resende R.R. (2016). Exploring the cell signaling in hepatocyte differentiation. Cell Signal..

[21] Wang P., Xu J., Wang Y., Cao X. (2017). An interferon-independent lncRNA promotes viral replication by modulating cellular metabolism. Science.

[22] Liu C.X., Chen L.L. (2022). Circular RNAs: Characterization, cellular roles, and applications. Cell.

[23] Ran F.L., Xu C., Li J., Wang H., Liu H., Yu Z., Zhao Z. (2023). Whole-transcriptome sequencing of phagocytes reveals a ceRNA network contributing to natural resistance to tuberculosis infection. Micro Pathog..

[24] Song Y.X., Sun J., Zhao J., Yang Y., Shi J., Wu Z., Chen X., Gao P., Miao Z., Wang Z. (2017). Non-coding RNAs participate in the regulatory network of CLDN4 via ceRNA mediated miRNA evasion. Nat. Commun..

[25] Lao Q.F., Zhang Q.Q., Qiao Z.P., Li S.L., Liu L., Martin F.L., Pang W.Y. (2022). Whole transcriptome sequencing and competitive endogenous RNA regulation network construction analysis in benzo[a]pyrene-treated breast cancer cells. Sci. Total Environ..

[26] Han L., Hao P., Wang W., Wu Y., Ruan S., Gao C.H., Tian W., Tian Y., Li X., Wang L. (2023). Molecular mechanisms that regulate the heat stress response in sea urchins (*Strongylocentrotus intermedius*) by comparative heat tolerance performance and whole-transcriptome RNA sequencing. Sci. Total Environ..

[27] Chen J.Q., Zhang S., Tong J.Y., Teng X.J., Zhang Z.Y., Li S., Teng X.H. (2020). Whole transcriptome-based miRNA-mRNA network analysis revealed the mechanism of inflammation-immunosuppressive damage caused by cadmium in common carp spleens. Sci. Total Environ..

[28] Gao Y.N., Wang Z.W., Yang X., Wang J.Q., Zheng N. (2023). Aflatoxin M1 and ochratoxin A induce a competitive endogenous RNA regulatory network of intestinal immunosuppression by whole-transcriptome analysis. Sci. Total Environ..

[29] Salmena L., Poliseno L., Tay Y., Kats L., Pandolfi P.P. (2011). A ceRNA hypothesis: The Rosetta Stone of a hidden RNA language?. Cell.

[30] Livak K.J., Schmittgen T.-D. (2001). Analysis of relative gene expression data using real time quantitative PCR and the 2(-Delta Delta C(T)) Method. Methods.

[31] Abbas Raza S.H., Abdelnour S.A., Alhumaidi Alotaibi M., AlGabbani Q., Naiel M.A.E. (2022). MicroRNAs mediated environmental stress responses and toxicity signs in teleost fish species. Aquaculture.

[32] Zheng W.W., Lv X., Xin S., Sun Y., Xu T. (2023). Long noncoding RNA TARL promotes antibacterial activity and prevents bacterial escape in *Miichthys miiuy* through suppression of TAK1 downregulation. Sci. China Life Sci..

[33] Song Y., Zheng W., Xin S., Pan J., Yang L., Sun Y., Xu T. (2023). Long noncoding RNA LTCONS6801 up-regulates TBKl mediated antiviralinnate immunity in miiuy croaker, *Miichttys miiuy*. Fish Shellfish Immunol..

[34] Holbrook J., Lara-Reyna S., Jarosz-Griffiths H.H., McDermott M.F. (2019). Tumour necrosis factor signalling in health and disease. F1000Research.

[35] van Loo G., Bertrand M.J.M. (2023). Death by TNF: A road to inflammation. Nat. Rev. Immunol..

[36] Gomez G.D., Balcázar J.L. (2008). A review on the interactions between gut microbiota and innate immunity of fish. FEMS Immunol. Med. Microbiol..

[37] Takeda K., Akira S.Z. (2004). TLR signaling pathways. Semin. Immunol..

[38] Lu X.B., Lu X., Zeng J., Jia K., Yi M. (2021). Antiviral activities of sea perch type I and type IIIFNs against RGNNV and their different roles inantigen presentation. Aquaculture.

[39] Johansson M.W. (1999). Cell adhesion molecules in invertebrate immunity. Dev. Comp. Immunol..

[40] Ding X.F., Xiang S.L. (2018). Endocytosis and human innate immunity. J. Immunol. Sci..

[41] Xu W.H., Dong J., Dai Y., Zhao Y., Qin Q., Huang X. (2024). Establishment of an LMBV-infection model in zebrafish larvae and its application in studying virus-host interactions. Aquaculture.

[42] Xu W., Zhang Z., Lai F., Yang J., Qin Q., Huang Y., Huang X. (2023). Transcriptome analysis reveals the host immune response upon LMBV infection in large mouth bass (*Micropterus salmoides*). Fish Shellfish Immunol..

[43] Gong Z.H., Fang F., Guo X.L., Guo C.F., Wang N., Wang L., Wang C.W., Hu G.B., Zhou B., Chen S.L. (2026). De novo establishment of a stable brain cell line from *Cephalopholis sonnerati* (tomato grouper) and transcriptomic dissection of its response to nervous necrosis virus (NNV) infection. Fish Shellfish Immunol..

[44] Peng Y., Han B., Zhang K., Tang P., Zhang Y., Ji J., Yin S., Ning X. (2023). ceRNA network mediated by lncRNA-miRNA-mRNA of *Pelteobagrus fulvidraco* plays a dual function of immunity and lipid metabolism in response to *Aeromonas veronii* infection. Aquaculture.

[45] Cai X., Lymbery A.J., Armstrong N.J., Gao C.B., Ma L., Li C. (2022). Systematic identification and characterization of lncRNAs and lncRNA-miRNA-mRNA networks in the liver of turbot (*Scophthalmus maximus* L.) induced with *Vibrio anguillarum*. Fish Shellfish Immunol..

[46] Niu J.J., Sun M.M., Li Z.Y., Wang Z.Y., Kong M., Wang Y.F., Song J.Q., Zhang Q.Q., He Y., Qi J. (2022). Whole transcriptome analysis provides new insight on immune response mechanism of golden pompano (*Trachinotus ovatus*) to *Amyloodinium ocellatum* infestation. Aquaculture.

[47] Ou J.T., Chen H., Luan X.Q., Ju R., Sun Y., Zhang B.H., Bian Y.X., Meng Y.S., Ji H., Wang Z.S. (2022). Leveraging lncRNA-miRNA-mRNA network to reveal anti-*Spiroplasma eriocheiris* infection mechanisms in *Macrobrachium nipponense*. Aquaculture.

[48] Chiang Y.H., Wu Y.C., Chi S. (2017). Interleukin-1b secreted from betanodavirus-infected microglia caused the death of neurons in giant grouper brains. Dev. Comp. Immunol..

[49] Liang K., Zhang M., Liang J., Zuo X., Jia X., Shan J., Li Z., Yu J., Xuan Z., Luo L. (2024). M1-type polarized macrophage contributes to brain damage through CXCR3.2/CXCL11 pathways after RGNNV infection in grouper. Virulence.

[50] Jiang X.Y., Chen Y., Ni J.J. (2025). Mechanism of miR-93 Regulating Immune-mediated Traumatic Brain Injury Through Activation of PI3K/AKT Pathway. Curr. Biotech..

[51] Schneeberger S., Kim S.J., Geesdorf M.N., Friebel E. (2025). Interleukin-12 signaling drives Alzheimer’s disease pathology through disrupting neuronal and oligodendrocyte homeostasis. Nat. Aging.

[52] Zhang Z., Zhang C. (2025). Regulation of cGAS-STING signalling and its diversity of cellular outcomes. Nat. Rev. Immunol..

[53] Abdelrahman A., Negroni C., Sahm F., Adams C.L., Urbanic-Purkart T., Khalil M., Vergura R., Morelli C., Hanemann C.-O. (2022). miR-497 and 219 in blood aid meningioma classification. J. Neuro-Oncol..

[54] Shan C.X., Wang J., Hai Q., Wang B., Kang W., Luo Z., Li Y., Liu X., Liu H., Liu Z. (2025). Study on immune difference of rainbow trout (*Oncorhynchus mykiss*) infected with infectious hematopoietic necrosis virus (IHNV) in two cultured water temperatures. Aquaculture.

