# Supplementary material for: LncRNA–miRNA–mRNA Regulatory Network Reveals Potential Immune Responses in Larval Tomato Hind (Cephalopholis sonnerati) Infected with RGNNV
Source: Biology (Basel). 2026 Aug 12;15(16):1379. doi: 10.3390/biology15161379 (PMC13510180; doi:10.3390/biology15161379)
Supplement: Supplementary file 1 [file biology-15-01379-s001.zip › biology-4429844-supplementary.pdf]

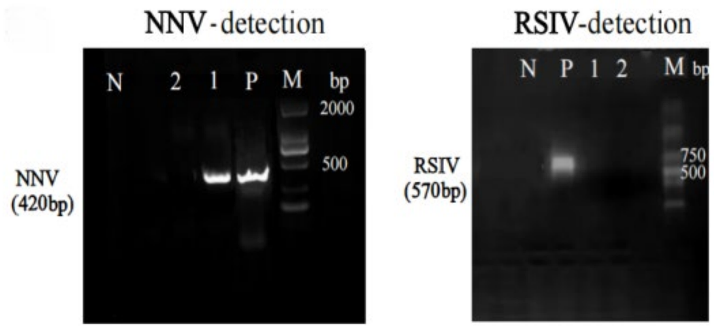

Figure S1. The virus detection in *C. sonnerati* brain tissue by PCR. Note: Photomicrographs of agarose gel electrophoresis after PCR amplification of NNV and RSIV. M, DNA marker; P, positive control; N, negative control; 1, infected samples; 2, healthy samples.

Table. S1 Sequences of primers used in experiment

Primers Name	Sequence of Primers(5′ — 3′)
RGNNV-F	CGTGTCAGTCATGTGTCGCT
RGNNV-R	CGAGTCAACACGGGTGAAGA
RSIV-F	CTCAAACACTCTGGCTCATC
RSIV-R	GCACCAACACATCTCCTATC
MSTRG.18695.1-F	GTTTAAAAGGGGGAACTCCA
MSTRG.18695.1-R	TTGTTGGGACAGACATTGGT
MSTRG.4342.33-F	GGCTTTGCCGCTTAAATTAT
MSTRG.4342.33-R	CACAGAGAGAGAACCAAAGAATG
MSTRG.1324.7-F	ATACGCTGATACCCCTGACC
MSTRG.1324.7-R	GGTAAAAGCTACAAAAAATCCG
MSTRG.5309.15-F	TAGCTGGACTGAGGGATGC
MSTRG.5309.15-R	CCCTTTAGGGTGGGAGAGAT
MSTRG.3629.2-F	AACATTCCCTTTGCCCTACA
MSTRG.3629.2-R	TGGCAAAGCGTGTTTTCTAC
circ_027622-F	GACACTGGTTCAACCCTCCT
circ_027622-R	ATGGCCAACTGCCTCTTTC
circ_026498-F	GGCCCTGATCACATGAAAAC
circ_026498-R	GGCTTGATGCACCACTCTGT
circ_016447-F	GCAGCGACCTATGTTCAGAA
circ_016447-R	GAACTCCTGATGGGTCCAGA

circ_003942-F	TCCAGAGAGGTGGTCTCCA
circ_003942-R	GTTCCACACCATATCTCTGCT
circ_029253-F	GCGACCAGTACCCTAACCT
circ_029253-R	CTTGCGCTGCTAATTTTCT
miR-462-F	CGGTAACGGAACCCATAATG
miR-1-3p-F	CGTGGAATGTAAAGAAGTATGGAT
miR-731-3p-F	CCGGGAATTTCGTGTCAGA
miR-222a-5p-F	GCGGCTCAGTAGTCAGTGTAGA
miR-15a-F	AGCAGCACATAATGGTTTGTG
miR-TY-R	GCTGTCAACGATACGCTACG
β-actin-F	TGGCATCACACCTTCTACAATGAG
β-actin-R	TCACACCATCACCAGAGTCCAT
U6-F	CTCGCTTCGGCAGCACA
U6-R	AACGCTTCACGAATTTGCGT

Table. S2 Data statistics of miRNA sequencing

Sample	Raw reads	Clean reads	Mapped(%)	N(%)	GC(%)	Q20(%)	Q30(%)
C_1	25175845	22564813	0.694	0.006009	43.75	99.44	97.48
C_2	24961502	20771575	0.704	0.006073	42.3	99.29	97.09
C_3	23159913	19786820	0.724	0.00521	43.53	99.52	97.67
NNV_1	12694892	11078679	0.741	0.00635	43.82	99.45	97.15
NNV_2	27096937	23884735	0.724	0.053504	42.89	99.56	97.89
NNV_3	31448066	28143412	0.734	0.003148	42.74	99.62	98.21

Table. S3 Data statistics of lncRNA sequencing

Sample	Reads No.	Bases (bp)	Q30 (bp)	N (%)	GC (%)	Q20 (%)	Q30 (%)
C_1	114102156	17229425556	16313194777	0.001603	48.76	98.08	94.68
C_2	125067444	18885184044	17992771310	0.001641	48.14	98.34	95.27
C_3	129793518	19598821218	18674807991	0.001621	49.29	98.32	95.29
NNV_1	148731192	22458409992	21400019451	0.001616	49.03	98.36	95.29
NNV_2	142117970	21459813470	20451221118	0.001615	48.34	98.36	95.30
NNV_3	174783932	26392373732	25186765024	0.001641	49.48	98.39	95.43

Table. S4 Statistics after lncRNA data filtering

Sample	Clean Reads No.	Clean Data (bp)	Clean Reads %	Clean Data %
C_1	112516546	16944784047	98.61	98.35
C_2	123769322	18629726202	98.96	98.65
C_3	128412256	19274077137	98.94	98.34
NNV_1	147236846	22162652319	99.00	98.68
NNV_2	140748970	21154736682	99.04	98.58
NNV_3	172983732	25988363839	98.97	98.47

Table. S5 Data statistics of circRNA sequencing

Sample	Anchors Reads	Map Reads	Map Reads (%)	Unique Reads	Unique Reads (%)	Multiple Reads	Multiple Reads (%)
C_1	39999040	33875890	84.69	6725192	16.81	27150698	67.88
C_2	38080460	30604579	80.36	6665577	17.50	23939002	62.86
C_3	45380812	37824771	83.35	6701469	14.77	31123302	68.58
NNV_1	59968354	38978203	64.99	8301500	13.84	30676703	51.15
NNV_2	43764654	34101164	77.92	7425176	16.97	26675988	60.95
NNV_3	83420136	46844290	56.16	10341157	12.40	36503133	43.76
